# Diagnosing Post-Polio Syndrome in the Elderly, a Case Report

**DOI:** 10.3390/geriatrics2020014

**Published:** 2017-04-30

**Authors:** Morolake Amole, Nadine Khouzam-Skelton

**Affiliations:** 1 Department of Internal Medicine, Morsani College of Medicine, University of South Florida, Tampa, FL 33612, USA; N.Khouzamskelton@va.gov; 2 Internal Medicine, James A Haley Veterans’ Hospital, Tampa, FL 33612, USA

**Keywords:** neurology, ambulatory care, comorbidity, primary care, geriatrics

## Abstract

Poliomyelitis is a disorder of the nervous system caused by an enterovirus. There are many survivors who, years later, develop a little-understood condition called Post-polio syndrome. Post-polio syndrome is a group of delayed sequalae of polio infection that can cause paralysis and bulbar symptoms in patients with a history of polio infection who have had a prolonged symptom-free period, often greater than two decades. Diagnosis of post-polio syndrome is difficult in the geriatric population because many of the symptoms overlap with other disease processes affecting older individuals. An extensive workup is necessary to exclude more concerning etiologies. Furthermore, several symptoms can be attributed to normal ageing. We present the case of an elderly patient with a history of poliomyelitis and multiple comorbidities who presented with complaints of weakness and fatigue.

## 1. Case Presentation

A 78-year-old male with a past medical history of hypertension, coronary artery disease status post myocardial infarction requiring stent placement, asthma, gastroesophageal reflux disease, and bulbar poliomyelitis presented to our Primary Care clinic for evaluation of worsening fatigue, exertional shortness of breath, dysphagia, chest tightness and generalized weakness.

The patient reported a diagnosis of poliomyelitis in 1956 after noticing flu-like symptoms and weakness. His course was complicated by dysphagia requiring tracheostomy placement, but no iron lung therapy was required. He reported appropriate recovery from his condition with few noticeable sequelae.

Vitals signs were within normal limits. Physical exam revealed an elderly male, alert, oriented, in no acute distress and with non-labored respirations. Neurological exam revealed mild bilateral upper extremity weakness. Sensation and reflexes were intact; positional and balance testing were normal and there were no cranial nerve abnormalities. Remaining cardiopulmonary, abdominal, musculoskeletal and skin exams were within normal limits. Routine blood testing revealed no abnormalities.

More extensive outpatient workup was initiated to elucidate possible etiologies of the patient’s symptoms. High Resolution CT Scan showed eventration and elevation of the right hemidiaphragm but no evidence of honeycombing, ground-glass opacification, suspicious lung nodules, bronchiectasis or bronchial wall thickening. Pulmonary Function testing was performed and results showed very mild restriction with a total lung capacity of 79%. A sleep study was also ordered and revealed mild obstructive sleep apnea.

Electrocardiogram revealed sinus rhythm and no ST-T wave abnormalities. Stress Echocardiogram showed an ejection fraction of 70%, no signs of ischemia and a non-reversible infarction in the basal inferolateral region that appeared unchanged from prior stress testing. Esophagogastroduodenoscopy (EGD) was also performed to further investigate the patient’s complaints of dysphagia. EGD showed a hiatal hernia in the esophagus as well as an esophageal schatzki’s ring requiring balloon dilation.

A diagnosis of post-polio syndrome was made given the patient’s distant history of poliomyelitis, mostly negative multi-system workup and presenting signs. Auto-pap was issued to the patient for the treatment of sleep apnea. Physical therapy was recommended with an emphasis on the avoidance of overexertion.

## 2. Discussion

### 2.1. Poliomyelitis

Poliomyelitis is caused by an enterovirus transmitted fecal-orally. Once inside the body, the virus can cross into the central nervous system (CNS) and affect the motor neurons of the spinal cord, brainstem and motor cortex, leading to paralytic poliomyelitis. Polio infection initially presents with malaise, fatigue, fever, sore throat, nausea and vomiting. Symptoms can then progress over the course of several days to include myalgias and muscle stiffness/spasms. In some patients, symptoms progress even further to paralysis.

The type of paralysis produced depends upon the portion of the CNS affected. For instance, spinal polio is caused by viral infiltration of the anterior horn of the spinal cord, and bulbar polio is caused by viral damage of the bulbar portion of the brainstem. Spinal poliomyelitis leads to asymmetric paralysis of the limbs due to spinal cord motor neuron damage. Bulbar polio, a significantly more rare form of poliomyelitis, affects the cranial nerves and thus the muscles they supply leading to respiratory difficulty as well as trouble with chewing, swallowing, and controlling facial movements [1].

### 2.2. Post-Polio Syndrome

Post-polio syndrome is a phenomenon that is characterized by muscle weakness and fatigue in patients with a history of poliomyelitis with complete recovery and who have had a long symptom-free period. In such patients, the key to diagnosis involves the history of polio, the presence of classic symptoms of motor neuron disease and the exclusion of other diagnoses [2]. The exact prevalence of post-polio syndrome is not known. According to the National Institute of Neurological Disorders and Stroke (NINDS), researchers have estimated that post-polio syndrome affects approximately 25%–40% of polio survivors.

The exact pathophysiology of post-polio syndrome is unknown, however, there are several theories that have been proposed. One theory is that during the acute infection, excess damage of motor neurons leads to the development of collateral motor neurons. Over time, it is impossible to maintain innervation and capillarisation of these compensatory motor neuron sprouts. The result is muscle weakness, muscle pain and easy fatigability. Another theory is persistence of polio infection. Several studies have reported the presence of poliovirus genome fragments in the cerebrospinal fluid (CSF) of patients with post-polio syndrome. A third theory is that there is a delayed immune response to the poliovirus, leading to chronic inflammation and thus persistent symptoms of poliomyelitis. This theory proposes treatment with immune modulators as means of combating the chronic inflammation [3].

Another proposed theory is that muscle weakness and fatigability may be the result of the normal ageing process causing symptoms in patients with post-polio syndrome [1]. It is possible that most of the symptoms experienced by this cohort of patients (elderly patients with a history of poliomyelitis infection) may be, mostly or in part, due to senescence.

Several proposed diagnostic criteria exist for post-polio syndrome, but most are based upon the criteria proposed by Halstead in the 1991 paper *Assessment and differential Diagnosis for Post-Polio Syndrome*. Criteria for the diagnosis of post-polio syndrome are as follows: (1) Prior diagnosis of polio must be confirmed; (2) There must be a period of functional and neurological stability; (3) There must be the onset of new neurological symptoms like weakness or fatigue; and (4) There must be an attempt to exclude of other medical diagnoses that may cause similar symptoms [4]. 

No specific mode of treatment exists. Treatment is mainly supportive, focusing on physical therapy and palliation. Physical therapy is used to build endurance but also focuses on avoiding overuse. Other therapies are aimed at improving quality of life by adding aids such as walkers. There is little in the way of pharmacologic treatment for this condition. Psychotherapy may also be beneficial given the significant psychological impact that poliovirus as well as its sequalae have on patients [1].

### 2.3. Primary Care Management of Post-Polio Syndrome in the Geriatric Population with Comorbidities

Post-polio syndrome is a fascinating disorder for the geriatric population. Its prevalence now in the United States can be correlated with 1950s epidemics. However, it is a difficult diagnosis in the ageing population because many of the classic symptoms of the disorder overlap with other disease processes affecting older populations, thus necessitating the need for extensive outpatient testing to exclude other plausible diagnoses. In patients with a history of heart disease, cardiac testing may be necessary to exclude acute coronary syndrome as the etiology of exertional shortness of breath and fatigue. This is even more important if the patient is female or has a history of diabetes mellitus, because these groups often present atypically. Electrocardiogram, echocardiogram, exercise or nuclear stress testing and possibly even heart catheterization may be necessary to further evaluate the condition [5].

Pulmonary etiologies must also be explored, even more so if the patient has a prior history of lung disease. In these instances, shortness of breath could be more attributable to decreased lung capacity as opposed to residual diaphragmatic paralysis from post-polio syndrome. Therefore, pulmonary function testing, chest imaging (high resolution CT imaging) or polysomnography may be necessary to evaluate complaints of breathing difficulty, fatigue and generalized weakness.

If the patient reports swallowing difficulty, it may be important to rule out gastroesophageal pathologies such as stricture or reflux prior to equating these symptoms to the prior history of bulbar polio. Consider esophagogastroduodenoscopy to further evaluate for anatomic abnormalities. It is also important to exclude other neurologic pathologies such as Parkinson’s disease, Amyotrophic lateral sclerosis or multiple sclerosis, as these symptoms also cause neuromuscular deficits similar to post-polio syndrome. Electromyography, lumbar puncture and MRI may be necessary. Electromyography is particularly helpful because findings can suggest where certain prior disease-affected muscle groups are located.

Excluding age as a cause of symptoms is very difficult, as there is much symptom overlap. If decline appears to be age-appropriate, then some symptoms may be related to ageing. There is little harm in attributing part of the disease process to age, as treatment of age-related decline in function is akin to treatment of post-polio syndrome.

Post-polio syndrome is an important primary care topic because its diagnosis requires extensive testing that is often prompted by the primary care specialist. Recognizing the signs and symptoms of the syndrome as well as a remote history of poliomyelitis is necessary [6].

## 3. Conclusions

Post-polio syndrome is an interesting disorder characterized by new neuromuscular deficits that present years after the resolution of a polio infection. There are several diagnostic criteria, but one of the most important is the exclusion of other possible diagnoses. Post-polio syndrome diagnosis is even more difficult in the elderly population due to the presence of multiple comorbid conditions. Extensive testing may be necessary for an appropriate diagnosis in this population.
